# Comparison of health care utilization among patients affiliated and not affiliated with healthcare professionals in China

**DOI:** 10.1186/s12913-020-05895-y

**Published:** 2020-12-03

**Authors:** Yafei Si, Zhongliang Zhou, Min Su, Han Hu, Zesen Yang, Xi Chen

**Affiliations:** 1 School of Risk & Actuarial Studies & CEPAR, The University of New South Wales, 223 Anzac Parade, Sydney, NSW 2052 Australia; 2 UNSW Ageing Futures Institute, The University of New South Wales, 223 Anzac Parade, Sydney, NSW 2052 Australia; 3 School of Public Policy and Administration, Xi’an Jiaotong University, No. 28 Xianning West Road, Xi’an, 710049 Shaanxi China; 4 School of Public Administration, Inner Mongolia University, No. 235 College Road, Hohhot, 010021 Inner Mongolia China; 5 Department of Political Science, Tsinghua University, Qinghua Yuan Street, No.1 Haidian District, Beijing, 100084 China; 6 School of Government, Sun Yat-Sen University, No.135, Xingangxi Road, Guangzhou, 510275 Guangdong China; 7 Department of Health Policy and Management, Yale School of Public Health, 60 College Street, New Haven, CT 06520 USA; 8 Department of Economics, Yale University, 60 College Street, New Haven, CT 06520 USA

## Abstract

**Background:**

Doing “more” in healthcare can be a major threat to the delivery of high-quality health care. It is important to identify the supplier-induced demand (SID) of health care. This study aims to test SID hypothesis by comparing health care utilization among patients affiliated with healthcare professionals and their counterpart patients not affiliated with healthcare professionals.

**Methods:**

We used coarsened exact matching to compare the health care utilization and expenditure between patients affiliated and not affiliated with healthcare professionals. Using cross-sectional data of the China Labour-force Dynamics Survey (CLDS) in 2014, we identified 806 patients affiliated with healthcare professionals and 22,788 patients not affiliated with healthcare professionals. The main outcomes were outpatient proportion and expenditure as well as inpatient proportion and expenditure.

**Results:**

The matched outpatient proportion of patients not affiliated with healthcare professionals was 0.6% higher (*P* = 0.754) than that of their counterparts, and the matched inpatient proportion was 1.1% lower (*P* = 0.167). Patients not affiliated with healthcare professionals paid significantly more (680 CNY or 111 USD, *P* < 0.001) than their counterparts did per outpatient visit (1126 CNY [95% CI 885–1368] vs. 446 CNY [95% CI 248–643]), while patients not affiliated with healthcare professionals paid insignificantly less (2061 CNY or 336 USD, *P* = 0.751) than their counterparts did per inpatient visit (15583 CNY [95% CI 12052–19115] vs. 17645 CNY [95% CI 4884–30406]).

**Conclusion:**

Our results lend support to the SID hypothesis and highlight the need for policies to address the large outpatient care expenses among patients not affiliated with healthcare professionals. Our study also suggests that as the public becomes more informed, the demand of health care may persist while heath care expenditure per outpatient visit may decline sharply due to the weakened SID. To address misbehaviors and contain health care costs, it is important to realign provider incentives.

**Supplementary Information:**

**Supplementary information** accompanies this paper at 10.1186/s12913-020-05895-y.

## Background

In the health care setting, “more” is not always better. Instead, doing “more” can be a major threat to the delivery of high-quality health care [1]. Increasing concerns have arisen about provider misbehavior because of their advantage of medical information and distorted financial incentives to provide costlier but unnecessary care, namely supplier-induced demand (SID), which is not aimed at improving health and general well-being of patients [2–4].

SID leads to overuse of healthcare, which is defined as the provision of health services that patients would not need or reject if they had full information or were fully informed [2–4]. Several methods have been advised to identify SID in health care. First, variation in physician income was used to test for induced behavior by examining the association between physician competition and health care utilization, and it was hypothesized that more intense competition would lead to fewer patients and hence an increase in SID [5–11]. Second, changes in physician fees were used to identify SID, mostly based on the target income hypothesis [12–15]. Finally, variation in patient information is important to test SID by identifying the effect of medical information on health care utilization [16, 17]. These studies suggest that medical information and physician incentives are important determinants of health care demand and health care expenditure. However, more work is needed before we can conclude about the economic importance of SID.

As a key part of health literacy, medical information helps patients understand health care service to make more informed choices [17, 18]. Health literacy concerns the knowledge and competences for people to make complex health decisions, and has received special attention in recent years, and it has been found associated with improved self-reported health, lower healthcare costs, more healthcare knowledge, shorter hospitalization, and reduced health care service use [18, 19]. Acquiring sufficient medical information allows patients to comprehend health care service and make informed choices while healthcare professionals would induce patients to use more by providing recommendations [20–22]. However, as for themselves, well-trained healthcare professionals well understand the problems they are looking to resolve; they can be aware of all potential medications for treatment and their potential side effects; they are regarded as the most informed patients [17, 23]. As the most informed patients, designating consumption of healthcare professionals as the gold standard does not imply that this is the proper standard for appropriate and effective health care [4, 17, 21]. However, their demand for health care can be an important benchmark to judge SID in the absence of well-established cost-benefit or risk-benefit analysis to assess the value of health care services.

A study in *NEJM* in 1974 firstly compared surgery rates for lawyers, businessmen, and ministers with those of physicians and found self-reported surgery rates to be equal or higher among healthcare professionals [16]. The same approach using survey data was adopted with more extensive controls, including income, insurance coverage, and self-reported health status, but they also find higher use among healthcare professionals [2]. In a more recent survey in Switzerland, healthcare professionals have much lower surgery rates than did the general population [17]. Less health service is provided to healthcare professionals than to others who lack the same medical knowledge but have similar healthcare demand and socioeconomic status [2, 16, 17, 24].

Previous research shared special interest in the amount of supplier-induced demand, while its economic consequence, another aspect of SID, is understudied although important. China unveiled its ambitious health system reform with the goal of providing affordable and equitable basic health care coverage for all by 2020 [25]. However, much of the government spending, health insurance funds, and out-of-pocket health care expenditure are likely captured by providers in the form of higher income and profits if the core culprit of rapidly increasing health care expenditure is the predominant fee-for-service payment system in China as well as other countries worldwide, driven by the overuse of examinations and drugs [25–27]. Empirical evidence of SID is difficult to obtain but crucial to advance health policy. We attempt to contribute to the literature on supplier-induced demand hypothesis by comparing the health care utilization among patients affiliated and not affiliated with healthcare professionals in China using national representative data.

## Methods

### Study setting and period

The study setting is China, all 29 out of 34 provinces excluding Hong Kong, Macau, Taiwan, Tibet and Hainan. We used the China Labor-force Dynamics Survey, which facilitates the study by covering a series of topics, such as family, education, employment, and health. Generally, China primarily use a “Fee-For-Service” payment method in health care, where health services are unbundled and paid separately. The “Fee-For-Service” payment scheme in health systems is frequently criticized for incentivizing providers to induce unnecessary demand with a higher quantity, especially on drugs and examinations with high profit margins. Analysis of this study was conducted from May 2017 to December 2017.

### Study design and data source

We used coarsened exact matching (CEM) to directly compare health care utilization and expenditure between patients affiliated and not affiliated with healthcare professionals. With no applicable policy changes or other forms of natural experiments, quasi-experimental matching methods become a viable strategy for causal inference. Prevalent matching methods, such as propensity score matching, often improve balance between the treated and control groups while leaving balance worse for some other variables. In other words, there is no guarantee of any level of overall imbalance reduction in any given data set [28]. However, CEM makes sure that the imbalance between the matched treated and control groups will not be larger than the ex-ante user choice. Improvements in the bound on balance for one covariate can be studied and improved in isolation as it will have no effect on the maximum imbalance of each of the other covariates [29]. King et al. (2011) further demonstrate that CEM dominates other matching methods in its ability to reduce imbalance, model dependence, estimation error, bias, variance, mean square error, and other criteria [30].

The comparison method in this study is distinct. In the absence of well-established cost-benefit or risk-benefit analysis to assess the value of health care services, the demand of health care for healthcare professionals can be regarded as an important benchmark to judge SID. In addition, we only matched patient information, which helps mitigate the concern over demand side driven healthcare utilization and reassures us the existence of SID.

The data were drawn from the China Labor-force Dynamics Survey (CLDS) conducted in 2014. CLDS is an open-access database and the first national longitudinal social survey on labor force in China, covering a series of topics, such as demographic characteristics, family, education, employment, work history, income, migration, and health (http://css.sysu.edu.cn/) [31, 32]. A multistage stratified cluster random sampling method was used, and the subjects of CLDS were the laborers (all family members aged 15–64) randomly selected from 29 provinces in China. The survey was conducted every 2 years and has accumulated three waves of data now (2012, 2014, and 2016). All investigators were trained before investigation and were monitored during the investigation. Computer-assisted personal interviewing (CAPI) technology was adopted to control data quality. The study was performed in 2017 when we used the available 2014 wave for analysis, in which more than 800 investigators collected 401 village questionnaires, 14,214 family questionnaires and 23,594 individual questionnaires.

Occupation information on each family member was collected in CLDS, and the occupation was classified using code in the fifth National Census. Following previous studies, our analysis defined patients affiliated with healthcare professionals as patients who were also healthcare workers, or patients with at least one family members who were healthcare professional (see healthcare professional list in Additional file 1: Appendix 1). Patients not affiliated with healthcare professionals were defined as patients who were not healthcare worker and at the same time had no family members who were healthcare professional. Finally, we identified 806 individuals affiliated with healthcare professionals and 22,788 individuals not affiliated with healthcare professionals for analysis.

### Variables

The CLDS collected information on outpatient use in the 2 weeks preceding the survey and inpatient use in 1 year preceding the survey to measure heath care utilization to avoid the recall bias in retrospective investigation [33]. Therefore, we generated four outcome variables, outpatient proportion (0-No, 1-Yes), outpatient expenditure (continuous variable measured by CNY), inpatient proportion (0-No, 1-Yes), and inpatient expenditure (continuous variable measured by CNY), for analysis.

A series of socio-demographic variables that might be associated with health care utilization was considered for inclusion in the matching. The variables were chosen based on a literature review and data availability (see detailed definition in Table 1) [4, 8–10]. We have a set of general variables including a health status indicator (self-reported question, 1-Healthy, 0-Fair/Unhealthy), coverage of health insurance (1-Yes, 0-No), living in urban or rural areas (1-Urban, 0-Rural), age (1-Ages equal and over 60, 0-Aged less 60), gender (1-Male, 0-Female), educational attainment (0-Primary school, 1-Middle school, 2-High school and above), access to healthcare (log of time to the nearest medical facilities) and economic status (log of household consumption per capita) in the matching for health care utilization. In addition, we have outpatient hospital tier (0-Primary, 1-Non-primary), inpatient hospital tier (0-Primary, 1-Secondary, 2-Tertiary) and inpatient reason (0-Else, 1-Disease, 2-Rehabilitation, 3-Fertility) for expenditure analysis.

**Table 1:** Variable definition and description

Variables	Definition	Affiliated		Not affiliated		Overall	
		N	%	N	%	N	%
Economic status ^a^	Log expenditure, mean (S.D.)	9.4	(1.0)	9.1	(1.0)	9.1	(1.0)
Gender	Male	440	54.6	11,830	51.9	12,270	52.0
	Female	366	45.4	10,957	48.1	11,323	48.0
Age	> = 60	694	88.6	19,010	87.5	19,704	87.5
	< 60	89	11.4	2726	12.5	2815	12.5
Health status	Healthy	270	33.5	8697	38.2	8967	38.0
	Fair/Unhealthy	536	66.5	14,090	61.8	14,626	62.0
Insurance	Insured	568	85.5	17,083	92.8	17,651	92.6
	Uninsured	96	14.5	1321	7.2	1417	7.4
Urban	Rural	306	38.0	14,126	62.0	14,432	61.2
	Urban	500	62.0	8662	38.0	9162	38.8
Access ^a^	Log time, mean (S.D.)	1.8	(0.8)	1.9	(0.8)	1.9	(0.8)
Education	Illiteracy	150	18.7	8351	36.8	8501	36.1
	Primary school	195	24.3	7545	33.2	7740	32.9
	Middle school	197	24.5	3974	17.5	4171	17.7
	High school and above	261	32.5	2854	12.6	3115	13.2
Outpatient level ^b^	Primary ^c^	23	60.5	725	67.1	748	66.9
	Non-primary	15	39.5	355	32.9	370	33.1
Inpatient level ^b^	Primary ^d^	11	22.9	302	23.1	313	23.1
	Secondary	34	70.8	924	70.6	958	70.6
	Tertiary	3	6.3	83	6.3	86	6.3
Inpatient reason ^b^	Else	4	8.3	90	6.9	94	6.9
	Disease	34	70.8	977	74.6	1011	74.5
	Rehabilitation	6	12.5	158	12.1	164	12.1
	Fertility	4	8.3	84	6.4	88	6.5

^a^Economic status is the natural logarithm of household consumption expenditure per capita per year; Access is the natural logarithm of time to go to the nearest medical institution (minutes)

^b^Individuals having outpatient visit or inpatient visit are summarized with outpatient level, inpatient level and inpatient reason

^c^Primary hospital for out visit includes village clinic/private clinic, township hospital and community healthcare center, while non-primary hospital for out visit includes the second level hospitals and above

^d^Primary hospital for in visit includes village clinic/private clinic, township hospital and community healthcare center

### Analysis and interpretation

We employed the coarsened exact matching (CEM) to better balance distributions of the covariates between the comparison groups and thereby reduce biases [28, 29, 34, 35]. A key property of CEM, comparing with propensity score matching (PSM), is that CEM fixes the maximum imbalance through an ex ante choice specified by the user, i.e., the user decides how the observed characteristics are to be coarsened. The user does not need to further conduct balance checking or restrict data to common support as required by PSM [28, 29, 34–36]. The matching approach helped to identify the counterparts for patients affiliated with healthcare professionals, based upon the observable pre-treatment characteristics. The general covariates were included in matching for health care utilization and we further included the hospital tier in the matching for per-outpatient expenditure and the hospital tier and inpatient reason in the matching for yearly inpatient expenditure. Overall, we carried out three coarsened exact matching processes in the study.

After the matching, 7722 patients not affiliated with healthcare professionals and 677 patients affiliated were identified for further analysis in health care utilization, 387 patients not affiliated with healthcare professionals and 32 patients affiliated were identified for further analysis in per-outpatient expenditure, and 195 patients not affiliated with healthcare professionals and 31 patients affiliated were identified for further analysis in yearly inpatient expenditure. The balance check (Additional file 1: Appendix 2) is reported to confirm that there is no statistical significance between the two groups.

Theoretically, with everything else equal, patients affiliated with healthcare professionals may use less healthcare and incur lower healthcare costs than patients not affiliated due to more information or higher health literacy. The difference in outcomes between the matched groups were regarded as supplied-induced demand and were accessed using 2-tailed t-tests and a significance threshold of *P* < 0.05. We adjusted the health care expenditure based on the average exchange rate in 2014 (100 USD = 614.28 CNY). Furthermore, we checked the robustness of our results using weighted regression analysis. All analyses were performed in Stata version 13.0 (Stata Corp LP, College Station, Texas, USA).

## Results

In the study, we identified 806 patients affiliated with healthcare professionals and 22,788 patients not affiliated. The basic information of their characteristics is showed in Table 1. Overall, there are 51.9% male respondents, and 87.5% of respondents are aged over 60 among patient not afflicated with healthcare professionals, slightly lower than those of patients affiliated with healthcare professionals. We find that patients not affiliated with healthcare professionals are more likely to be healthy (38.2%), to have medical insurance (92.8%), to live in a rural area (62.0%), and to be less educated than patients affiliated with healthcare professionals. Furthermore, patients not affiliated with healthcare professionals are more likely to use outpatient services at a primary hospital (67.1%) than patients affiliated with healthcare professionals. We identified the same trend in inpatient health care utilization, without much difference in inpatient reason.

In Table 2, the initial comparison of outcomes was conducted prior to matching. Almost 4.8% (95% CI 4.5–5.0) of patients not affiliated with healthcare professionals used outpatient care in the last 2 weeks, a little higher than that for patients affiliated with healthcare professionals, i.e. 4.7% (95% CI 3.3–6.2). Meanwhile, 5.8% (95% CI 5.5–6.1) of patients not affiliated with healthcare professionals utilized inpatient care in the past year, a figure lower than 6.1% (95% CI 4.4–7.7) for patients affiliated. Furthermore, outpatient expenditure per visit for patients not affiliated with healthcare professionals is 1200 CNY (95% CI 1034–1367), much higher than 479 CNY (95% CI 278–681) for patients affiliated. However, yearly average inpatient expenditure for patients not affiliated with healthcare professionals is 13172 CNY (95% CI 11580–14765), which is lower than 15256 CNY (95% CI 6634–23878) for patients affiliated.

**Table 2:** Health care utilization and expenditure before matching

Outcomes	Group	N	%	95% confidence interval	
				Lower	Upper
Outpatient visit	Affiliated	806	4.7	3.3	6.2
	Not affiliated	22,788	4.8	4.5	5.0
Inpatient visit	Affiliated	806	6.1	4.4	7.7
	Not affiliated	22,788	5.8	5.5	6.1
Outpatient expenditure, mean (S.D.)	Affiliated	479	(613)	278	681
	Not affiliated	1200	(2773)	1034	1367
Inpatient expenditure, mean (S.D.)	Affiliated	15,256	(29693)	6634	23,878
	Not affiliated	13,172	(29365)	11,580	14,765

In Table 3, we compared health care utilization of the two groups across several socioeconomic factors. We find that gender was associated with outpatient and inpatient health care utilization of patients not affiliated with healthcare professionals (*P* < 0.001) but not patients affiliated (*P* = 0.932, *P* = 0.505). It is similar with living in the urban/rural areas (*P* < 0.001, *P* = 0.001 vs. *P* = 0.590, *P* = 0.904). Educational attainments were associated with outpatients and inpatients health care utilization of patients not affiliated with healthcare professionals (*P* < 0.001, *P* < 0.001). However, educational attainments were associated with inpatients health care utilization (*P* = 0.060) but not outpatient health care utilization of patients affiliated (*P* = 0.294). Health status was significantly associated with outpatient health care utilization (*P* < 0.001, *P* < 0.001) and inpatient health care utilization (*P* < 0.001, *P* < 0.001) of both patients affiliated and not affiliated with healthcare professionals. Finally, fewer socio-demographic factors were associated with health care expenditure of the two groups.

**Table 3:** Comparing health care utilization and expenditure across socio-demographic groups

Variables	Outpatient visit (%)		Inpatient visit (%)		Outpatient expenditure (CNY), mean (S.D.)				Inpatient expenditure (CNY), mean (S.D.)			
	Not affiliated	Affiliated	Not affiliated	Affiliated	Affiliated		Not affiliated		Affiliated		Not affiliated	
Gender												
Female	5.3	4.8	6.5	6.6	1900	(8286)	421	(636)	12,588	(30427)	13,464	(29970)
Male	4.2	4.6	5.1	5.5	2196	(6875)	552	(595)	13,976	(27846)	17,765	(29886)
*P*-value ^a, b^	< 0.001	0.932	< 0.001	0.505	0.533		0.521		0.399		0.626	
Education												
Illiteracy	6.4	6.0	7.9	9.3	1546	(6487)	547	(845)	12,809	(32741)	10,531	(21576)
Primary school	4.2	6.7	5.3	6.7	2593	(8408)	565	(458)	13,149	(25734)	19,000	(32797)
Middle school	3.7	3.6	4.5	7.1	1631	(4930)	140	(198)	13,711	(24162)	20,900	(39989)
High school and above	2.9	3.5	2.9	3.1	3481	(13552)	603	(778)	15,330	(27865)	6738	(4037)
*P*-value	< 0.001	0.294	< 0.001	0.060	0.066		0.382		0.895		0.646	
Age												
< 60	4.6	4.6	5.1	5.8	1886	(6980)	439	(619)	12,486	(22571)	14,679.5	(27806)
> =60	6.1	6.7	9.8	9.0	2855	(11250)	698	(582)	17,570	(48419)	19,912.5	(40800)
*P*-value	0.001	0.379	< 0.001	0.232	0.143		0.349		0.015		0.657	
Health												
Fair/Unhealthy	9.1	10.0	11.2	10.7	2243	(8115)	545	(670)	14,971	(33168)	19,768	(37726)
Healthy	2.1	2.1	2.5	3.7	1440	(6484)	338	(460)	8206	(13161)	8940	(9297)
*P*-value	< 0.001	< 0.001	< 0.001	< 0.001	0.129		0.339		< 0.001		0.217	
Insurance												
No	5.1	5.5	6.6	6.2	2023	(7365)	488	(599)	13,357	(30247)	17,583	(33547)
Yes	5.2	3.1	5.1	7.3	2653	(12229)	380	(539)	15,737	(26064)	7000	(4637)
*P*-value	0.880	0.338	0.034	0.674	0.520		0.767		0.532		0.490	
Urban												
Rural	5.2	5.2	6.2	6.2	1642	(6651)	566	(633)	12,007	(30256)	12,172	(18667)
Urban	4.0	4.4	5.2	6.0	2823	(9512)	416	(605)	15,489	(27396)	17,107	(34859)
*P*-value	< 0.001	0.590	0.001	0.904	0.018		0.465		0.043		0.583	

^a^Chi-2 test was used for dummy variable.

^b^Univariate ANOVAs was employed for continuous variables

**Fig. 1:**
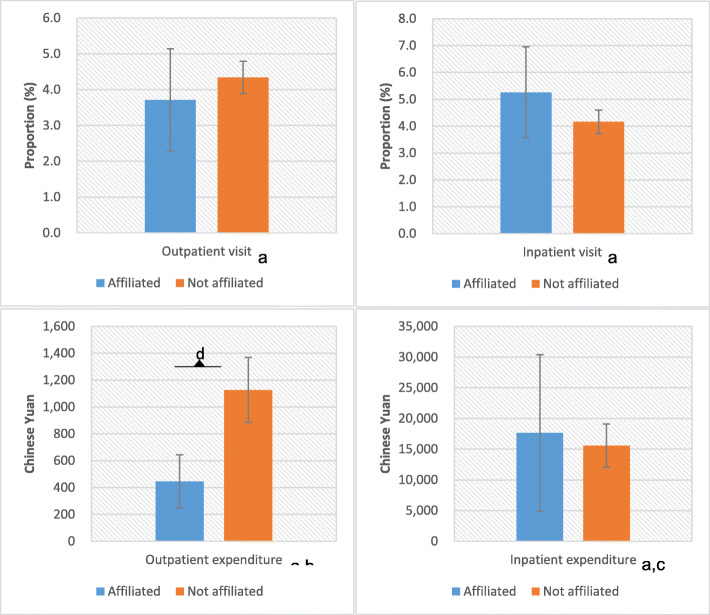
Comparison of health care utilization and expenditure among patients affiliated and not affiliated with healthcare professionals. Notes: ^a^Coarsened exact matching was used including health status indicator, health insurance, living in the urban or rural area, age, gender, educational level and economic status in the matching for health care utilization. ^b^Outpatient level was further added in the matching for per-outpatient expenditure. ^c^Inpatient level and inpatient reason were further added in the matching for yearly inpatient expenditure. ^d^*p* < 0.001

The final results were displayed after controlling for possible confounding factors. Figure 1 indicates that the outpatient proportion of patients not affiliated with healthcare professionals is 0.6% (*P* = 0.754) higher than that of patients affiliated (4.3% [95% CI 3.9–4.8] vs. 3.7% [95% CI 2.3–5.1]), while the inpatient proportion of patients not affiliated with healthcare professionals is 1.1% (*P* = 0.167) lower than that of patients affiliated (4.2% [95% CI 3.7–4.6] vs. 5.3% [95% CI 3.6–7.0]), but the differences are not statistically significant. Moreover, patients not affiliated with healthcare professionals paid significantly more (680 CNY or 111 USD, *P* < 0.001) than patients affiliated did per outpatient visit (1126 CNY [95% CI 885–1368] vs. 446 CNY [95% CI 248–643]). However, patients not affiliated with healthcare professionals paid less (2061 CNY or 336 USD, *P* = 0.751) than patients affiliated did in the last year but with no statistical significance (15584 CNY [95% CI 12052–19115] vs. 17645 CNY [95% CI 4884–30406]).

## Discussion

In our study including 23,594 respondents in China, we used coarsened exact matching to compare the health care utilization and expenditure between patients affiliated and not affiliated with healthcare professionals to estimate the supplier-induced demand of health care in China. We found little evidence of statistical difference in the outpatient proportion or the inpatient proportion between the two groups, suggesting that patients affiliated and not affiliated with healthcare professionals have comparable demand of health care. The exception is that we exhibited a significant higher (680 CNY or 111 USD, or on average 56.7% of outpatient expenditure) per-outpatient health care expenditure in patients not affiliated with healthcare professionals.

Our result highlighted strong economic significance of supplier-induced demand on outpatient health care expenditure, indicating that patients not affiliated with healthcare professionals paid more for unnecessary service and treatment compared with patients affiliated with healthcare professionals. Healthcare professionals are often blamed for the high cost of health care services as they are often able to induce patients to consume more health care than necessary [4, 33]. For instance, studies in developed countries also have illustrated that physicians tend to perform more cesarean delivery in response to declining fertility, treat more intensively when their incomes are adversely affected by fee-reduction policies, and prescribe more medications to patients [10, 37–39].

In this study, however, patients not affiliated with healthcare professionals paid less (2061 CNY or 336 USD) than affiliated patients did in inpatient health care in the past year, and the difference is not statistically significant. There are some plausible explanations for the seemingly conflicting findings between outpatient spending and inpatient spending. Firstly, inpatient expenditure is measured as the total expenditure of inpatient health care in the past year, rather than the per-inpatient expenditure. Patients affiliated with healthcare professionals have a slightly higher inpatient proportion than that of patients not affiliated, which may contribute to their higher total inpatient expenditure. Secondly, the total inpatient expenditure is usually positively associated with the number of inpatient health care service. Therefore, predicted higher numbers of inpatient use in patients affiliated with healthcare professionals could have led to the higher inpatient expenditure in the last year. Thirdly, it is possible that patients not affiliated with healthcare professionals would mobilize their social resources or social capital to seek inpatient care in China [40–42]. Therefore, more studies are needed to investigate these.

The strength of this study builds on the direct comparison of health care utilization between patients affiliated and not affiliated with healthcare professionals after controlling for confounding factors. The demand of health care for healthcare professionals can be regarded as an important benchmark to judge SID in the absence of well-established cost-benefit or risk-benefit analysis to assess the value of health care services. However, research on health care service consumption by patients affiliated with healthcare professional is scarce, though they may best recognize risks and benefits of health care service. Compared with existing literature, we employ large-scale national representative data. In addition, our defined medical information provision allows diffusion within household, which enables us to consider the fact that healthcare professionals as informed individuals often help their household members make better health care decisions. Finally, we examine SID in the largest developing county as compared to most previous studies in developed countries. By measuring the differences in outcomes among patients affiliated and not affiliated with healthcare professionals, this approach provides a direct channel to estimate SID.

We are confident that the method is effective although there is concern that healthcare professionals working in different departments acquired different medical information, with the potential to create significant information barriers among themselves. However, differences in medical information does not invalidate the approach for reasons stated below. Firstly, information always diffuses efficiently and spills across departments in the same hospital as healthcare professionals work in a shared environment, which empowers themselves. Secondly, favoritism exchanges are essential in Chinese setting. Most cross-cultural comparisons in the management literature illustrate that the Chinese, including healthcare professionals, are collectivists with respect to their working motivation and achieving personal goals by working with members of their ego-centered trust networks [40–43]. Finally, cooperative game works in healthcare setting. Healthcare professionals have to interact in repeated games to maximize their own benefits, the case of which gradually leads to a cooperative game without additional harms [44, 45].

Our study has been carried out in China where healthcare professionals are paid on a fee-for-service basis and where financial budget constraints prevent residents from obtaining basic health care [27, 46, 47]. The characteristics of system determine that healthcare professionals have strong motivation to provide more care to compensate their expected low wage. Recently, China advanced significantly to address problems of unaffordable coverage and illness-associated impoverishment by offering substantial public funding [25, 26]. However, considering the significant effect of SID on the outpatient health care, incentivizing physicians to provide less avoidable care may effectively reduce health care costs in China. Our study suggests that the demand of health care may sustain as the public becomes more informed while the health care expenditure of outpatient may decrease sharply. Recognizing the effect of medical information on health, researchers have been focusing on the development of cost-effective interventions to improve health literacy or to limit the problems posed by low health literacy. Previous studies indicated that the overall level of health literacy among Chinese residents was relatively low [48]. Therefore, health promotion and education may play an important role in patient empowerment, which in turn leads to better self-management of medical conditions, ultimately lowering costs of care [33, 48, 49].

### Limitations

In this study, the observations of CLDS are labours, who generally have a lower outpatient proportion and inpatient proportion compared with the old and children, and thus we have a slightly smaller sample size to compare their health care expenditure after the matching. Our estimates would be more precise and possibly a multilevel statistical analysis would provide more insights with larger sample size. In addition, the results cannot be interpreted as causal effect regarding the cross-sectional study design. Ideally, more precise estimation of SID should involve direct comparison of the two groups infected with the same disease. Importantly, more socio-demographic analysis can be performed with a larger sample size to quantify cost variations in age, health insurance coverage, public/private hospital, and geographical area.

## Conclusion

Patients not affiliated with healthcare professionals have nearly equal demand of health care compared with patients affiliated with healthcare professionals. However, we find that patients not affiliated with healthcare professionals exhibited a significant higher (680 CNY or 111 USD, or on average 56.7% of outpatient expenditure) per-outpatient health care expenditure than their counterpart group. Our findings support the supplier-induced demand hypothesis and highlight the need to better understand the rapidly rising health care expenditure in China, a driver of overuse of healthcare. Our study suggests that as the public becomes more informed, the demand of health care may persist while health care expenditure per outpatient visit may decrease sharply due to the decline in SID. Therefore, providing requisite medical information and promoting health education for patients is important for lowering the ever-increasing costs of healthcare. Importantly, as significant misbehaviors performed by healthcare professionals, further study to understand causes why physicians prescribed unnecessary services and treatment is pressing. One frequently discussed core culprit is the predominant fee-for-service payment system, and thus China must reform its incentive structures for healthcare providers, improve the governance of hospitals, and institute a stronger regulatory system. The gap in outpatient care expenditure in China is large, and creating incentives among physicians to provide less care may work well to reduce health care costs.

## Supplementary Information

**Additional file 1 **: **Appendix 1.** Code of occupation classification in CLDS. **Appendix 2.** Balance check after matching.

## Data Availability

Data used in this paper are from the China Labor-force Dynamics Survey (CLDS) by the Center for Social Science Survey at Sun Yat-sen University in Guangzhou, China. The opinions are the author’s alone. The data is open for the public (please refer to http://css.sysu.edu.cn).

## Abbreviations

SID: Supplier-induced demand
CLDS: China labour-force dynamics survey
CEM: Coarsened exact matching
CAPI: Computer-assisted personal interviews
CNY: Chinese Yuan
USD: United State dollar
