# Supplementary material for: Comparison of health care utilization among patients affiliated and not affiliated with healthcare professionals in China
Source: BMC Health Serv Res. 2020 Dec 3;20:1118. doi: 10.1186/s12913-020-05895-y (PMC7713311; doi:10.1186/s12913-020-05895-y)
Supplement: Supplementary file 1 — Additional file 1 : Appendix 1. Code of occupation classification in CLDS. Appendix 2. Balance check after matching. [file 12913_2020_5895_MOESM1_ESM.docx]

Appendix 1 Code of occupation classification in CLDS

| 1-9  1-91  1-92  1-93  1-94  1-95  1-96  1-97  1-98  1-99 | Professional and technical personnel Physician Traditional Chinese medical doctor Chinese and western medicine doctors National physician Public health doctor Pharmaceutical personnel Medical Technician Nurse Other professional |
| --- | --- |

**Notes:** The table shows occupation code of medical professional and technical personnel section only.

Appendix 2 Balance check after matching

|  |  | Health care utilization | | |  | Outpatient expenditure | | |  | Inpatient expenditure | | |
| --- | --- | --- | --- | --- | --- | --- | --- | --- | --- | --- | --- | --- |
|  |  | Affiliated | Not affiliated | P-value |  | Affiliated | Not affiliated | P-value |  | Affiliated | Not affiliated | P-value |
| Economic status | Log expenditure | 9.18 | 9.18 | 0.760 |  | 8.98 | 8.94 | 0.746 |  | 9.43 | 9.26 | 0.206 |
| Gender | Male | 56.76 | 56.76 | 1.000 |  | 60.81 | 60.81 | 1.000 |  | 69.74 | 69.74 | 1.000 |
|  | Female | 43.24 | 43.24 |  |  | 39.19 | 39.19 |  |  | 30.26 | 30.26 |  |
| Age | >=60 | 96.54 | 96.54 | 1.000 |  | 96.44 | 96.44 | 1.000 |  | 86.46 | 86.46 | 1.000 |
|  | <60 | 3.46 | 3.46 |  |  | 3.56 | 3.56 |  |  | 13.54 | 13.54 |  |
| Health status | Health | 26.63 | 26.63 | 1.000 |  | 87.79 | 87.79 | 1.000 |  | 87.18 | 87.18 | 1.000 |
|  | Fair/Unhealthy | 73.37 | 73.37 |  |  | 12.21 | 12.21 |  |  | 12.82 | 12.82 |  |
| Insurance | Insured | 95.62 | 95.62 | 1.000 |  | 99.22 | 99.22 | 1.000 |  | 98.44 | 98.44 | 1.000 |
|  | Uninsured | 4.38 | 4.38 |  |  | 0.78 | 0.78 |  |  | 1.56 | 1.56 |  |
| Urban | Rural | 56.93 | 56.93 | 1.000 |  | 70.74 | 70.74 | 1.000 |  | 50.26 | 50.26 | 1.000 |
|  | Urban | 43.07 | 43.07 |  |  | 29.26 | 29.26 |  |  | 49.74 | 49.74 |  |
| Access | Log time | 1.79 | 1.79 | 0.967 |  |  |  |  |  |  |  |  |
| Education | Illiteracy | 27.84 | 27.84 | 1.000 |  |  |  |  |  |  |  |  |
|  | Primary school | 33.36 | 33.36 |  |  |  |  |  |  |  |  |  |
|  | Middle school | 20.89 | 20.89 |  |  |  |  |  |  |  |  |  |
|  | High school and above | 17.91 | 17.91 |  |  |  |  |  |  |  |  |  |
| Outpatient level | Primary |  |  |  |  | 72.77 | 72.77 | 1.000 |  |  |  |  |
|  | Non-primary |  |  |  |  | 27.23 | 27.23 |  |  |  |  |  |
| Inpatient level | Primary |  |  |  |  |  |  |  |  | 7.69 | 7.69 | 1.000 |
|  | Secondary |  |  |  |  |  |  |  |  | 89.23 | 89.23 |  |
|  | Tertiary |  |  |  |  |  |  |  |  | 3.08 | 3.08 |  |
| Inpatient reason | Else |  |  |  |  |  |  |  |  |  |  | 1.000 |
|  | Disease |  |  |  |  |  |  |  |  | 89.74 | 89.74 |  |
|  | Rehabilitation |  |  |  |  |  |  |  |  | 5.13 | 5.13 |  |
|  | Fertility |  |  |  |  |  |  |  |  | 5.13 | 5.13 |  |
| N |  | 677 | 7722 |  |  | 32 | 387 |  |  | 31 | 195 |  |

**Notes:** Economic status is the natural logarithm of household consumption expenditure per capita per year; Access is the natural logarithm of time to go to the nearest medical institution (minutes); Primary hospital for out visit includes village clinic/private clinic, township hospital and community healthcare center, while non-primary hospital for out visit includes the second level hospitals and above; Primary hospital for in visit includes village clinic/private clinic, township hospital and community healthcare center; Univariate ANOVAs was employed for continuous variables and chi-2 test was used for dummy variable.
